# Watchful Waiting Compared With Immediate Antibiotics for Urban Aboriginal and Torres Strait Islander Children With Uncomplicated Acute Otitis Media (WATCH): A Non‐Inferiority Randomised Controlled Trial

**DOI:** 10.5694/mja2.70260

**Published:** 2026-08-13

**Authors:** Jennifer S. Reath, Hasantha Gunasekera, Sanja Lujic, Amanda J. Leach, Letitia Campbell, Robyn Walsh, Tim Usherwood, Geoffrey K. Spurling, Claudette A. Tyson, Deborah A. Askew, Kelvin Kong, Chelsea J. Watego, Peter Morris, Wendy Hu, Penelope A. Abbott

**Affiliations:** ^1^ Western Sydney University Sydney New South Wales Australia; ^2^ University of Sydney Sydney New South Wales Australia; ^3^ UNSW Sydney Sydney New South Wales Australia; ^4^ Menzies School of Health Research Darwin Northern Territory Australia; ^5^ Kalwun Development Corporation Gold Coast Queensland Australia; ^6^ Children's Cancer Research Unit Sydney Children's Hospitals Network Sydney New South Wales Australia; ^7^ Queensland Centre of Excellence in Aboriginal and Torres Strait Islander Primary Health Care Brisbane Queensland Australia; ^8^ University of Queensland Brisbane Queensland Australia; ^9^ University of Newcastle Newcastle New South Wales Australia; ^10^ Macquarie University Sydney New South Wales Australia; ^11^ Queensland University of Technology Brisbane Queensland Australia; ^12^ Royal Darwin Hospital Darwin Northern Territory Australia

**Keywords:** anti‐infective agents, child health, ear diseases

## Abstract

**Objective:**

Determine whether watchful waiting is non‐inferior to immediate oral antibiotics for uncomplicated acute otitis media among urban Aboriginal and Torres Strait Islander children.

**Study Type:**

Non‐inferiority unblinded randomised controlled trial.

**Setting and Participants:**

Eight Aboriginal Medical Services across three Australian states and territories between 25 August 2014 and 2 June 2023. Children (aged 1.5–16 years) with type B tympanograms and bulging tympanic membrane or acute pain/irritability were randomised by site and age (1.5–6 years and 7–16 years), with stratification using randomly allocated, permuted blocks of four and six in length.

**Main Outcome Measures:**

Watchful waiting compared with immediate oral antibiotics using modified intention‐to‐treat (using only available data) and per‐protocol analyses of Day 7 clinical resolution with non‐inferiority threshold set at 10 percentage points.

**Results:**

Children were randomly allocated to watchful waiting (134), six of whom were lost to follow‐up or immediate antibiotics (129) with three lost to follow‐up. Resolution occurred in 57/106 (53.8%) watchful waiting and 68/113 (60.2%) immediate antibiotic group of those with complete Day 7 data (−6.4 percentage points difference; 90% confidence interval [CI], −17.4 to 4.6) (modified intention‐to‐treat analysis). Per‐protocol analysis similarly demonstrated reduced resolution in the watchful waiting group (49/97; 50.5%) compared with immediate antibiotics (67/112; 59.8%) with −9.3 percentage points difference (90% CI, −20.6 to 2.0). There was less Day 3 diarrhoea in watchful waiting (3/90; 3.3%) than in the immediate antibiotic group (13/93 [14.0%]; −10.7 percentage points difference; 95% CI, −18.6 to −2.7) but no other differences in vomiting, diarrhoea or rash (Days 3–14). Day 7 analgesia use was higher in the watchful waiting (53/107 [49.5%]) than immediate antibiotic group (37/116 [31.9%]; 17.6 percentage points difference; 95% CI, 4.9 to 30.1). There were no intervention‐related severe adverse events or perforations.

**Conclusion:**

Although our results are numerically similar to those reported in other low‐risk populations and no unexpected safety signals were observed in the watchful waiting arm, non‐inferiority was not established. Larger, adequately powered trials are required to determine whether watchful waiting is non‐inferior in this population.

**Trial Registration:**

Australian New Zealand Clinical Trials Registry ACTRN#12613001068752

## Plain Language Summary

**The:** Acute otitis media is a common reason for childhood antibiotic prescriptions. Aboriginal and Torres Strait Islander children are at high risk of complications, yet management evidence from urban settings, where most live, is lacking.

**The New:** Watchful waiting had slightly lower resolution and more analgesia use on Day 7, comparable with international studies of low‐risk children, although non‐inferiority was not demonstrated. There were no perforations or between‐group differences in Week 7 middle ear effusions. Parent/carers were equally satisfied with each option.

**The Implications:** Watchful waiting is acceptable management for urban Aboriginal and Torres Strait Islander children with uncomplicated acute otitis media.

## 1 Introduction

Acute otitis media is among the most common childhood infections and a frequent reason for prescribing antibiotics [1, 2, 3]. Mismanaged acute otitis media increases the risk of recurrent and persistent disease and lifelong impacts on hearing, auditory‐processing, learning, educational and employment outcomes [4]. Meta‐analyses of randomised controlled trials show earlier resolution and fewer symptoms with immediate systemic antibiotics prescription for acute otitis media [3]. However, benefits of immediate antibiotic treatment are small in magnitude and adverse effects including vomiting, diarrhoea and rash are more common [3, 5]. Antibiotic stewardship necessitates rationalisation of antibiotic therapy to those most likely to benefit [6]. Given these considerations, most international guidelines currently recommend watchful waiting (no immediate antibiotics) except for children at risk of complications [4, 7, 8, 9, 10, 11].

Aboriginal and Torres Strait Islander children in remote settings experience acute otitis media earlier, more often and with more complications than other children, so antibiotics are recommended in these settings [4]. However, most Aboriginal and Torres Strait Islander children live in urban areas [12], with lower rates of acute otitis media and its complications [13, 14]. Current Australian guidelines recommend watchful waiting for urban Aboriginal and Torres Strait Islander children who are at low risk of complications, but these recommendations are extrapolated from international studies, as no trials among this population have been conducted [4].

To our knowledge, the WATCH trial (Watchful Waiting for Aboriginal and Torres Strait Islander Children with Acute Otitis Media) was the first study investigating whether watchful waiting was non‐inferior to immediate antibiotics in this setting. We chose a non‐inferiority trial design as any benefit of watchful waiting would be from lower healthcare costs, less imposition on families and fewer antibiotic prescriptions, with decreased adverse effects and antibiotic resistance. Based on previous studies, we assumed a clinical improvement rate of 80% at 1 week [15] and specified a 10‐percentage point non‐inferiority threshold [15, 16].

## 2 Methods

This trial was registered with the Australian New Zealand Clinical Trials Registry on 24 September 2013 (ACTRN12613001068752). This article follows the relevant CONSORT guideline [17], with a checklist provided in Table S1.

### 2.1 Study Design

This study is a non‐inferiority, open‐label randomised controlled trial.

### 2.2 Setting and Participants

Partnering health services were all urban, including two Aboriginal Community Controlled Health Services in Sydney, one in each of the New South Wales Central Coast, Canberra, Townsville, Brisbane and the Gold Coast and one Indigenous health service in Brisbane. Partner sites received equipment, training and funding to employ site‐based research officers, most of whom identified as Aboriginal and/or Torres Strait Islander. Staff and members of community reference groups at most sites provided input to the research team (a mix of Aboriginal and non‐Aboriginal researchers) and guided research implementation within each community [16], thereby building cultural responsiveness.

Aboriginal and/or Torres Strait Islander children aged 18 months to 16 years (inclusive) with unilateral or bilateral acute otitis media without perforation, living in urban communities and who had not previously been recruited to the trial were eligible. We started recruitment with a lower age of 2 years but changed mid‐trial to 18 months for children with unilateral acute otitis media to improve recruitment rates, as this was consistent with guidelines [18].

Children were excluded from recruitment if they had taken antibiotics in the previous 4 days; were at high risk of chronic suppurative otitis media (residing in areas known to have prevalence greater than 4%); had grommets in situ or current or past tympanic membrane perforation; increased risk of complications, such as immunosuppression, genetic or chromosomal abnormality, cleft palate or mid‐face abnormalities; or systemic features that, in the view of the general practitioner, necessitated immediate antibiotic treatment.

Children attending participating services between 25 August 2014 and 2 March 2023 were screened by site‐based research officers trained in trial protocol [16], video‐pneumatic otoscopy and tympanometry. Children with a bulging tympanic membrane and/or ear pain (or irritability in pre‐verbal children) with a type B tympanogram were diagnosed with acute otitis media and informed about the trial. A general practitioner worked with the research officer to confirm the diagnosis, check for exclusion criteria and obtain informed, written consent from the parent/carer and older children [19]. Although the study protocol [16] stated nurse practitioners could replace general practitioners, no site employed a nurse practitioner in this role. Study assessments are described in Table S2. Adverse events were actively sought at predetermined timepoints. Trial assessment findings, including tympanograms, were incorporated into the child's medical record.

### 2.3 Interventions

All parent/carers were advised to use analgesia as needed. Parent/carers of children randomly allocated to watchful waiting were not given an immediate antibiotic prescription but were informed that antibiotics could be prescribed subsequently if required. Participants who were prescribed antibiotics after 48 h from recruitment were considered adherent with watchful waiting. Children randomly allocated to immediate antibiotics received an antibiotic script, which parents/carers were encouraged to fill as soon as possible. General practitioners chose the antibiotic and dose but were advised to follow Australian prescribing guidelines [18].

### 2.4 Outcomes

The primary outcome was the proportion of children with general practitioner‐determined clinical resolution on Day 7, defined as no pain (or irritability in pre‐verbal children), no fever (> 38°C), no bulging tympanic membrane and no complications.

Secondary outcomes are listed in Table 1 and results analysed with a null hypothesis of no difference between groups. Economic analysis will be reported in a separate publication.

**TABLE 1:** Secondary outcomes.

Outcome	Assessed by	When^a^
1. Proportion of children with resolution of signs of otitis media	Blinded otolaryngologist/paediatrician assessment of video‐otoscopy images and tympanometry traces taken by a research officer	Days 0 and 7
2. Proportion of children with middle ear effusion or tympanic membrane perforation and chronic suppurative otitis media	Blinded otolaryngologist assessment of video‐otoscopy images and tympanometry traces taken by a research officer	Week 7
3. Proportion of children with antibiotic prescriptions provided for the index acute otitis media at any time after recruitment	Medical record review by medical practitioner researcher and standardised parent/carer questionnaire administered by research officers	3 months
4. Parent/carer‐reported acute otitis media symptoms assessed by AOM‐SOS symptom questionnaire^b^	Administered by research officer	Days 3, 7 and 14
5. Use of analgesia for acute otitis media symptom relief from recruitment to Day 14 as assessed by standardised parent/carer questionnaire	Administered by research officer	Days 3, 7 and 14 and Week 7
6. Parent/carer satisfaction with acute otitis media treatment assessed by standardised parent/carer questionnaire with rating scale	Administered by research officer	Day 14

Abbreviation: AOM‐SOS, Acute Otitis Media—Severity of Symptoms Version 4.

^a^ Acceptable time windows: Day 3 (2–4); Day 7 (5–10); Day 14 (11–17) and Week 7 (6–8).

^b^ The Acute Otitis Media Faces Scale was planned to be included in this analysis; however, insufficient data were collected using this survey tool.

All timepoints had acceptability windows: Day 3 (2–4), Day 7 (5–10), Day 14 (11–17) and Week 7 (6–8).

### 2.5 Sample Size and Data and Safety Monitoring

A sample of 198 children per group had 80% power to demonstrate a 10‐percentage point non‐inferiority margin, assuming 80% resolution in the control arm (immediate antibiotics). The 10‐percentage point margin is an estimation of clinically significant difference [15]. Allowing for 20% loss to follow‐up, our recruitment target was 495 children. For quality assurance purposes, blinded otolaryngologists or paediatricians with expertise in otitis media reviewed video‐otoscopy images and/or tympanograms taken at Days 0 and 7 and at Week 7 from one or both ears of a subset of children. An independent Data Safety Monitoring Board (including the trial statistician) with pre‐determined stopping criteria reviewed unblinded data regularly [16].

Adverse events were captured in face‐to‐face or phone interviews on Days 3, 7 and 14 and on Week 7. Severity and causality were determined by the general practitioner and ‘trial‐related’ or ‘possibly related’ events were reviewed by an independent safety monitor who reported to the Data Safety Monitoring Board.

### 2.6 Sequence Generation

The general practitioner or research officer randomly allocated children to watchful waiting (intervention) or immediate antibiotics (control) using the National Health and Medical Research Council Clinical Trial Centre Interactive Voice Response System, with the formula set at trial commencement by the trial manager. Children were assigned 1:1, stratified by age (1.5–6 years and 7–16 years) and recruitment site, using randomly allocated, permuted blocks of four and six in length. The sequence was concealed, so the randomiser did not know the upcoming allocation, but it was not possible to blind families or research and clinic staff to allocation. Otolaryngologists and paediatricians reviewing a sub‐set of video‐otoscopy images and/or tympanograms were blinded to the child's allocation and the time data were collected.

### 2.7 Implementation

The site‐based research officer or general practitioner recorded the allocation in the child's study file and medical record and explained the allocated management to the parent/carer.

### 2.8 Statistical Methods

Consistent with our protocol plan, which was published before analysis [16], the primary outcome assessment included all children with Day 7 data. We compared trial arms using modified intention‐to‐treat and per‐protocol analyses to avoid the risk of finding non‐inferiority due to non‐adherence to either therapeutic arm [17]. The per‐protocol analysis compared only children in the immediate treatment group who received an antibiotic script within 48 h with only those in the watchful waiting group who did not receive an antibiotic script within 48 h—consistent with a watchful waiting approach [4, 7, 8, 11].

The non‐inferiority test was conducted using the Farrington–Manning method [20], with a one‐sided significance level of 0.05% and 90% two‐sided confidence intervals (CIs) for the difference in resolution rates between the watchful waiting and immediate antibiotic arms. Non‐inferiority was concluded if the lower bound of the 90% CI exceeded the non‐inferiority margin of −10 percentage points. Sensitivity analyses were conducted according to a range of assumptions regarding missing data. Planned analysis of resolution extrapolating from a Day 3 telephone interview was not undertaken due to lack of data. Post hoc analysis using a symptom‐based definition of resolution of acute otitis media enabled comparison of our findings with other published research [21]. Subgroup analyses compared outcomes for children with different baseline characteristics. For secondary outcomes, we used modified intention‐to‐treat analysis to examine differences between the two groups, using two‐sided *t*‐tests or χ^2^ tests as appropriate, reporting means or proportions and 95% CIs. There were no adjustments for multiple testing. Data were analysed using SAS version 9.4 (SAS Institute).

### 2.9 Ethics

From inception and throughout the research process, our approaches conformed with key ethical statements, guidelines and principles for research in Aboriginal and Torres Strait Islander health [19, 22, 23, 24, 25]. We report according to the CONSIDER statement (Table S3) [26].

The study was approved by the following ethics committees: Aboriginal Health and Medical Research Council Ethics Committee as lead Ethics Committee (938/13); Western Sydney University Human Research Ethics Committee (13/012032, H10369); Human Research Ethics Committee for the Northern Territory, Department of Health and Menzies School of Health (HOMER 13/2074); Metro South Human Research Ethics Committee (Queensland Department of Health; HREC/13/QPAH/366); and the University of Queensland Medical Research Ethics Committee (2013001093).

## 3 Results

**FIGURE 1:**
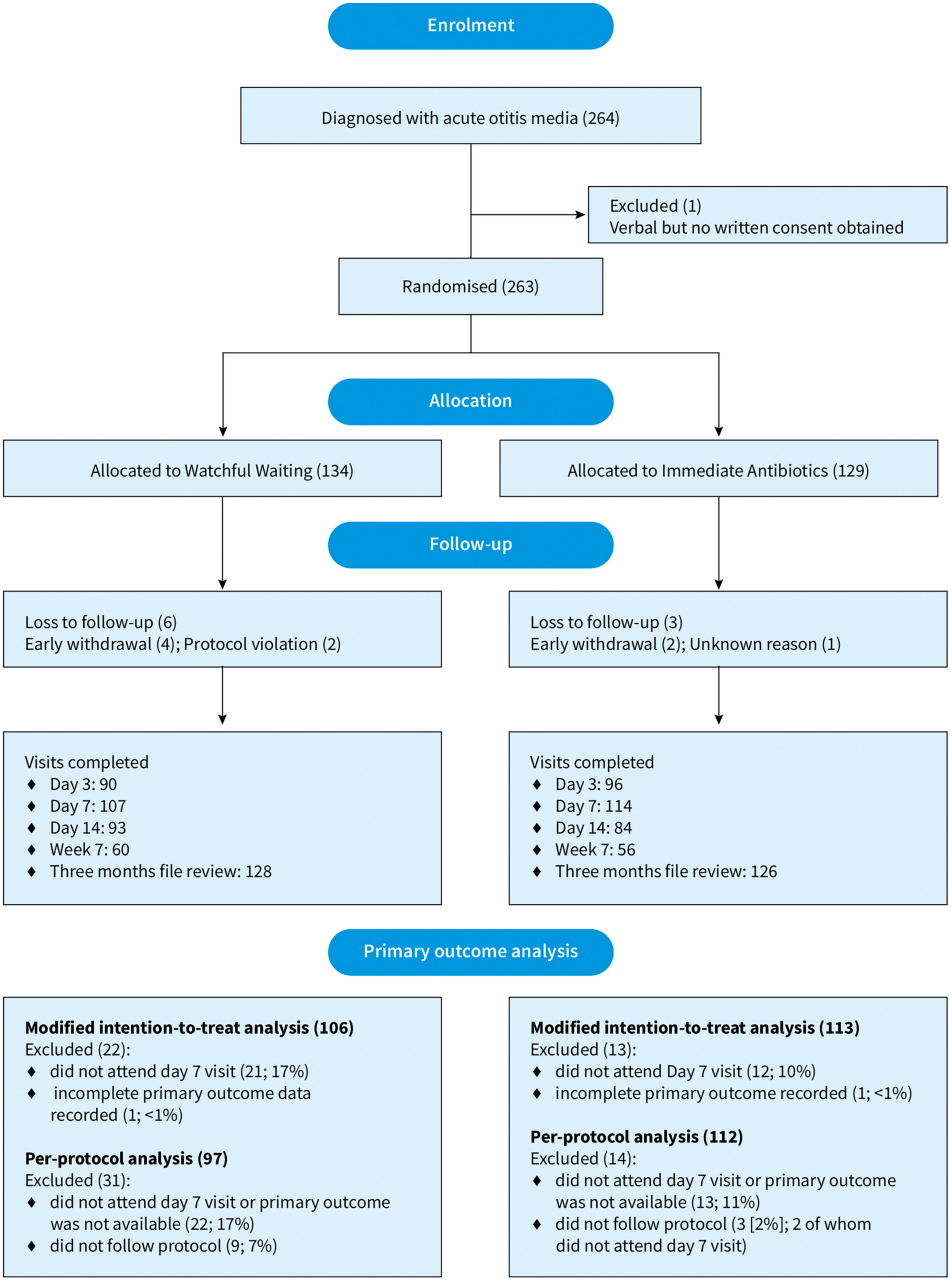
Flowchart of recruitment, exclusions and follow‐up.

Participant recruitment and flow are presented in Figure 1.

We randomly allocated 263 children (123 from one site and 140 from the other seven) between 8 September 2014 and 2 March 2023. Follow‐up data collection occurred between 12 September 2014 and 2 June 2023. Screening logs were not consistently recorded at all sites, so numbers screened and/or excluded are not reportable. Recruitment was greatly affected by COVID‐19 pandemic‐related service closures and restrictions in children with respiratory symptoms attending services. We closed the trial when funding ran out, with 128 children in the watchful waiting arm and 126 children in the immediate antibiotic arm group after exclusions and withdrawals. We had primary outcome data for 106/128 in the watchful waiting group and 113/126 in the immediate antibiotics group, providing a total of 219/254 (86.2%) children with data available for the primary outcome modified intention‐to‐treat analysis.

### 3.1 Baseline Data

Risk factors and symptoms were similar between groups (Table 2). Blinded review of all interpretable video‐otoscopy recordings (56) and 179 tympanograms (52 children had both measurements) identified three children, all randomly allocated to immediate antibiotics, who had ear‐canal discharge on Day 0. A sensitivity analysis excluding these children was conducted, as tympanic membrane perforation could not be ruled out.

**TABLE 2:** Baseline characteristics of trial participants by group allocation.

Child characteristics	Watchful waiting	Immediate antibiotics
Total number of participants^a^	128	126
Sex^b^		
Female	61/128 (47.7%)	57/126 (45.2%)
Male	67/128 (52.3%)	69/126 (54.8%)
Age (years)		
< 3	50/128 (39.1%)	52/126 (41.3%)
3 to < 6	48/128 (37.5%)	49/126 (38.9%)
6 to < 12	27/128 (21.1%)	20/126 (15.9%)
12–16	3/128 (2.3%)	5/126 (4.0%)
Household crowding (people per bedroom)		
> 2	9/120 (7.5%)	13/119 (10.9%)
≤ 2	111/120 (92.5%)	106/119 (89.1%)
History		
Previous ear infection	73/119 (61.3%)	76/114 (66.7%)
Previous grommets	5/117 (4.3%)	2/117 (1.7%)
Up to date immunisation	109/118 (92.4%)	113/117 (96.6%)
Child influenza vaccination (past 12 months)	22/113 (19.5%)	20/112 (17.9%)
Breastfeeding (ever)	75/116 (64.7%)	85/116 (73.3%)
Dummy use (ever)	66/115 (57.4%)	65/118 (55.1%)
Exposure to smoking (ever)	67/118 (56.8%)	72/117 (61.5%)
Unilateral compared with bilateral^c^		
Unilateral	58/126 (46.0%)	56/123 (45.5%)
Bilateral	68/126 (54.0%)	67/123 (54.5%)
Symptoms and signs		
General practitioner‐determined bulge of tympanic membrane	95/125 (76.0%)	101/120 (84.2%)
Irritability (infants aged < 3 years only)	38/45 (84.4%)	38/49 (77.6%)
Symptoms (‘a lot’)		
Tugging, rubbing	26/120 (21.7%)	29/122 (23.8%)
Ear pain	34/118 (28.8%)	36/116 (31.0%)
Crying	43/123 (35.0%)	29/122 (23.8%)
Irritable, fussy	48/123 (39.0%)	46/123 (37.4%)
Difficulty sleeping	38/122 (31.1%)	42/120 (35.0%)
Less playful or active	18/124 (14.5%)	14/122 (11.5%)
Eating less	25/124 (20.2%)	25/121 (20.7%)
Having fever	22/122 (18.0%)	22/122 (18.0%)
Vomiting	2/124 (1.6%)	3/121 (2.5%)
Having diarrhoea	0/123 (0%)	2/121 (1.7%)
Coughing	49/124 (39.5%)	50/121 (41.3%)
Runny nose	50/121 (41.3%)	51/121 (42.1%)
Rash	0/123 (0%)	2/120 (1.7%)
AOM‐SOS categorised		
Less symptomatic (score < 6)	81/124 (65.3%)	81/123 (65.9%)
More symptomatic (≥ 6)	43/124 (34.7%)	42/123 (34.1%)
AOM‐Faces Scale: rating of severity of symptoms right now		
1 = Not present/not a problem	19/125 (15.2%)	29/122 (23.8%)
2 = Hardly a problem	22/125 (17.6%)	21/122 (17.2%)
3 = Somewhat a problem	27/125 (21.6%)	20/122 (16.4%)
4 = Moderate problem	19/125 (15.2%)	20/122 (16.4%)
5 = Quite a bit of a problem	19/125 (15.2%)	19/122 (15.6%)
6 = Very much a problem	16/125 (12.8%)	7/122 (5.7%)
7 = Extreme problem	3/125 (2.4%)	6/122 (4.9%)

Abbreviations: AOM, acute otitis media; AOM‐SOS, Acute Otitis Media—Severity of Symptoms Version 4.

^a^ Denominators include only children for whom baseline data were available.

^b^ Parent/carer‐reported sex of child.

^c^ Where laterality was recorded.

In the immediate antibiotic arm, 92 (91.1%) of the 101 prescriptions recorded were for amoxicillin, six (5.9%) for amoxicillin with clavulanic acid and one each for cefuroxime, cephalexin, erythromycin and penicillin.

### 3.2 Outcomes and Estimation

Unless otherwise specified, all analyses are based on available data at the relevant timepoint.

#### 3.2.1 Primary Outcome

Resolution of acute otitis media on Day 7 was lower for the watchful waiting (57/106; 53.8%) than for the immediate antibiotic group (68/113; 60.2%). Modified intention‐to‐treat analysis did not demonstrate non‐inferiority, as the percentage point difference (−6.4) had wide CIs crossing the non‐inferiority margin of −10 percentage points (−17.4 to 4.6) (Table 3). Per‐protocol analysis similarly demonstrated lower resolution in watchful waiting (49/97; 50.5%) compared with immediate antibiotics (67/112; 59.8%) with −9.3 percentage points difference (90% CI, −20.6 to 2.0).

**TABLE 3:** Outcome and safety data analyses.

	Watchful waiting^a^	Immediate antibiotics^a^	Comparison between watchful waiting and immediate antibiotic groups	*p*
			Difference in percentage points (90% CI)	
Primary outcomes				
Total number of participants	128	126		
Number of participants with modified intention‐to‐treat analysis data	106/128 (82.8%)	113/126 (89.7%)		
General practitioner‐determined resolution	57/106 (53.8%)	68/113 (60.2%)	−6.4 (−17.4 to 4.6)	0.29^b^
Number who followed protocol	119/128 (93.0%)	123/126 (97.6%)		
Number with per‐protocol analysis data	97/119 (81.5%)	112/123 (91.1%)		
General practitioner‐determined resolution	49/97 (50.5%)	67/112 (59.8%)	−9.3 (−20.6 to 2.0)	0.46^b^
			**Difference in percentage points (95% CI)**	
Secondary outcomes				
1. Proportion of children with resolution of signs of otitis media at Day 7	13/63 (20.6%)	19/72 (26.4%)^c^	−5.8 (−20.0 to 8.5)	0.43
2. Proportion of children with middle ear effusion, tympanic membrane perforation and chronic suppurative otitis media at Week 7	Middle ear effusion: 16/36 (44.4%) Perforations/chronic suppurative otitis media: 0/36 (0%)	Middle ear effusion: 21/34 (61.8%) Perforations/chronic suppurative otitis media: 0/34 (0%)	−17.4 (−40.4 to 5.7)	0.15
3. Proportion of children with antibiotic prescriptions provided for the index acute otitis media at any time after recruitment to 3 months	26/128 (20.3%)	124/126 (98.4%)	**−78.1 (−85.4 to −70.8)**	**< 0.001**
	**Mean (SD)**	**Mean (SD)**	**Difference in mean scores (95% CI)**	
4. Parent/carer‐reported acute otitis media symptoms assessed by AOM‐SOS symptom questionnaire				
AOM‐SOS as a continuous variable				
Baseline	4.2 (2.8)	4.0 (2.8)	0.2 (−0.5 to 0.9)	0.60
Day 3	1.8 (2.3)	1.7 (2.3)	0.2 (−0.5 to 0.8)	0.67
Day 7	1.2 (1.8)	0.7 (1.4)	**0.5 (0.1 to 0.9)**	**0.024**
Day 14	0.6 (1.4)	0.4 (1.1)	0.2 (−0.2 to 0.6)	0.26
			**Difference in percentage points (95% CI)**	
5. Use of analgesia for acute otitis media symptom relief from recruitment to Day 14 as assessed by standardised parent/carer questionnaire				
Day 3	48/87 (55.2%)	44/94 (46.8%)	8.4 (−6.2 to 22.9)	0.26
Day 7	53/107 (49.5%)	37/116 (31.9%)	**17.6 (4.9 to 30.1)**	**0.007**
Day 14	20/92 (21.7%)	9/83 (10.8%)	10.9 (0.1 to 21.7)	0.05
Week 7	15/58 (25.9%)	19/55 (34.5%)	−8.7 (−25.6 to 8.2)	0.31
6. Parent/carer satisfaction with acute otitis media treatment assessed by standardised parent/carer questionnaire with rating scale at Day 14	89/90 (98.9%)	76/79 (96.2%)	2.7 (−2.1 to 7.4)	0.25
Adverse outcomes				
Specific symptoms^d^ on Day 3				
Vomiting	5/90 (5.6%)	4/93 (4.3%)	1.3 (−5.0 to 7.5)	0.69
Diarrhoea	3/90 (3.3%)	13/93 (14.0%)	**−10.7 (−18.6 to** − **2.7**)	**0.011**
Rash	4/89 (4.5%)	4/92 (4.3%)	0.1 (−5.8 to 6.1)	0.96
Specific symptoms^d^ on Day 7				
Vomiting	6/109 (5.5%)	7/116 (6.0%)	−0.5 (−6.6 to 5.6)	0.86
Diarrhoea	6/109 (5.5%)	8/116 (6.9%)	−1.4 (−7.7 to 4.9)	0.67
Rash	8/109 (7.3%)	5/114 (4.4%)	2.9 (−3.2 to 9.1)	0.35
Specific symptoms^d^ on Day 14				
Vomiting	4/93 (4.3%)	6/83 (7.2%)	−2.9 (−9.9 to 4.0)	0.40
Diarrhoea	3/93 (3.2%)	6/83 (7.2%)	−4.0 (−10.6 to 2.6)	0.23
Rash	4/93 (4.3%)	1/82 (1.2%)	3.1 (−1.7 to 7.8)	0.22

Abbreviations: AOM‐SOS, Acute Otitis Media Severity of Symptoms Version 4; CI, confidence interval; SD, standard deviation.

^a^ Denominators include only children for whom data were available at the relevant timepoint.

^b^ *p* for non‐inferiority.

^c^ One child in the immediate antibiotic arm was noted to have a perforation at Day 7 on blinded asynchronous expert review of tympanogram/video‐otoscopy traces.

^d^ Including those noting either ‘a little’ or ‘a lot’.

#### 3.2.2 Secondary Outcomes

In a sub‐group analysis (Table 3), resolution of acute otitis media determined by a blinded otolaryngologist and/or paediatrician review of video‐otoscopic images and tympanogram traces at Day 7 was similar in both trial arms. The proportion of children with Week 7 middle‐ear effusion was similar, with no tympanic membrane perforation or chronic suppurative otitis media observed. Watchful waiting was associated with significantly reduced antibiotic use up to 3 months from diagnosis. Acute Otitis Media—Severity of Symptoms (AOM‐SOS) Version 4 scores at Day 7 revealed that children in the watchful waiting arm had significantly more symptoms than those receiving immediate antibiotics. They also received more analgesia at Day 7. Parent satisfaction with treatment of their child's acute otitis media on Day 14 was high in both groups: 89/90 (98.9%) in the watchful waiting group and 76/79 (96.2%) in the immediate antibiotic group (2.7 percentage point difference; 95% CI, −2.1 to 7.4).

### 3.3 Ancillary Analyses

Sensitivity analyses of the primary outcome were conducted as specified in the published protocol [16] and showed non‐inferiority only under the assumption that all missing visits resolved in the watchful waiting arm and failed to resolve in the immediate antibiotic arm. Post hoc analysis of symptom resolution at Day 7 according to Hoberman's criteria [21] found the watchful waiting arm had a resolution rate more than 10 percentage points inferior to the immediate antibiotic arm (Table S4). However, 90% CIs included the non‐inferiority margin (watchful waiting 74/108 [68.5%] vs. immediate antibiotics 98/116 [84.5%]; risk difference, −16.0 percentage points; 90% CI, −25.2 to −6.8). Analyses of resolution of subgroups of children (unilateral disease, bilateral disease, highly symptomatic and less symptomatic) did not reveal significant differences between arms.

Time‐to‐event analyses and survival curves could not be generated due to the absence of precise resolution dates.

Four severe adverse events were reported at three study sites, comprising severe vomiting and diarrhoea (two children); severe vomiting related to concussion; and a severe rash. Only the child with the rash was in the watchful waiting group. All four severe adverse events were deemed unrelated to the trial by the treating general practitioner (3) or independent safety monitor (1). All resolved without sequelae.

## 4 Discussion

To our knowledge, this is the first trial to examine whether watchful waiting—the standard care for uncomplicated acute otitis media globally—is an acceptable option for urban Aboriginal and Torres Strait Islander children. We found clinical resolution at Day 7 in 57/106 (53.8%) in the watchful waiting and 68/113 (60.2%) in the immediate antibiotic trial arms. We did not demonstrate non‐inferiority of watchful waiting, as 90% CIs included the non‐inferiority margin. However, our point estimate was within the margin and the small difference in resolution rates we found is similar to those reported in other studies globally [21, 27]. Although our Day 7 resolution rate for children in the immediate antibiotic group (60.2%) was lower than that reported by Hoberman (80%) and Tahtinen (81%) [21, 27], Hoberman's definition of resolution was AOM‐SOS scores of 0 or 1 and Tahtinen's was a composite measure of improvement based on symptoms and otoscopic signs. Using Hoberman's definition, our resolution rates were similar. Children randomly allocated to watchful waiting received 80% fewer antibiotic scripts.

Our findings are comparable to other studies internationally and provide the only data examining this question among urban Aboriginal and Torres Strait Islander children, indeed, among any First Nations children globally. These data are long overdue, as previously guidelines have extrapolated from non‐Indigenous populations in high‐income countries or from trials conducted among children from remote settings, whereas most Aboriginal and Torres Strait Islander children live in urban settings [12].

Reported rates of symptoms and side effects were similar between study arms. At Day 7, we found a higher mean AOM‐SOS score among the watchful waiting group, although the difference was small (0.5/10), the change from baseline was unlikely to be appreciable to parents/carers [28] and the mean scores would have been classified as ‘asymptomatic’ in other studies [21]. Adverse events were minor, but we found slightly more diarrhoea in the immediate antibiotic group, consistent with other studies [21, 27]. There were no serious adverse events linked to watchful waiting.

National guidelines on management of acute otitis media recommend immediate antibiotics for Aboriginal and Torres Strait Islander children in remote settings [4]. These children have among the highest rates of otitis media, severe disease and complications in the world [2, 4, 29]. However, these guidelines recommend watchful waiting for Aboriginal and Torres Strait Islander children in urban settings based on data from other populations [4]. Our findings suggest that acute otitis media resolution among urban Aboriginal and Torres Strait Islander children is similar to that in other low‐risk populations. Furthermore, tympanic membrane perforations are less common than in remote settings [13, 14]. Although one child in the immediate antibiotic arm was noted on Day 7 blinded review to have a perforation, at Week 7, no mastoiditis, cholesteatoma, chronic suppurative otitis media or other perforations were identified through chart review or when 69 blinded review assessments of both ears were undertaken at Week 7.

In a randomised controlled trial of azithromycin compared with amoxicillin for acute otitis media in remote Aboriginal children, 4% had perforations at Day 11 [30] and, in the placebo group of the Hoberman trial, 5% had perforations and one child developed mastoiditis [21]. In our trial, at Week 7, middle ear effusion was as common in both groups, detected in at least one ear in 37/70 (52.9%) children on blinded review, similar to 54% at 6 weeks in a meta‐analysis examining the natural history of untreated otitis media [31], but higher than the 40% at about 3 weeks and 23% at about 3 months in a recent Cochrane review among low‐risk populations [3].

We have previously reported that watchful waiting was an acceptable approach for families of urban Aboriginal and Torres Strait Islander children when they are partners in decision‐making [32]. In this article, we add that parents/carers were equally satisfied with management approaches in each arm of the trial (Table 3).

The quality of randomised controlled trials in this setting must be judged by more than the outcome data [25]. Our process evaluation [33] reinforced the importance of Aboriginal and Torres Strait Islander insights, co‐design and co‐implementation throughout this trial. These were critical in ensuring that the research was a priority, interventions were acceptable, and trial conduct was culturally appropriate and responsive to communities [16, 33]. Aboriginal Medical Service‐based research officers led published sub‐studies and contributed to research demonstrating the value of these roles and the clinical support for ear health they provided throughout the trial [32, 34, 35, 36].

### 4.1 Limitations

Screening logs were not consistently maintained at all sites; hence, screening numbers cannot be reported nor prevalence of acute otitis media in these communities estimated. Recruitment was heavily impacted by COVID‐19, which prevented us from reaching our target sample size [16]. However, we randomly allocated 263 children, similar to the two major randomised controlled trials examining this question in high‐income non‐Indigenous settings: Hoberman (291) [21] and Tahtinen (322) [27]. Rare adverse effects of management in either arm may not have been detected. Unblinded allocation may have impacted on clinician and researcher assessment of participant outcomes. Our trial was pragmatic, designed to assess safety and non‐inferiority of watchful waiting in primary care. Therefore, general practitioners were free to choose antibiotics, and different team members assessed the child's progress at study visits, although more than 92/101 (90%) adhered to guideline recommendations for amoxicillin. We used a definition of resolution that included resolution of bulging, which was not used by other otitis media researchers, and may have contributed to lower than expected Day 7 resolution rates. The symptom tools we employed had not been previously used with Aboriginal and Torres Strait Islander populations [37].

Missing data may have reduced our estimation of difference in resolution if those allocated to watchful waiting dropped out due to treatment failure. However, the difference in loss to follow‐up was small and the per‐protocol analysis does not show different findings from the modified intention‐to‐treat analysis.

About half of our sample was recruited at one of the eight sites, introducing potential sampling bias. However, that site was a large service for this population in a major city and likely to be representative of urban Aboriginal and Torres Strait Islander children. We believe our pragmatic trial design promotes the generalisability of our findings to general practice by matching normal clinical practice in the management of acute otitis media.

### 4.2 Conclusion

To our knowledge, our trial is the only trial examining watchful waiting for acute otitis media among Aboriginal and Torres Strait Islander children, despite many trials globally comparing watchful waiting with immediate antibiotic treatment. Our results are numerically similar to those reported in other low‐risk populations with similar differences in resolution rates and side effects and no unexpected safety signals were observed in the watchful waiting arm.

Our survey found that parents/carers were highly satisfied with both management approaches, adding to previously reported interview findings that watchful waiting is an acceptable approach for families of Aboriginal and Torres Strait Islander children living in urban settings when they are partners in the decision‐making process [32]. Although our results lend support to current national guidelines [4] advising that Aboriginal and Torres Strait Islander children from urban settings with low‐risk of complications can be managed consistent with recommendations for other low‐risk children globally [4, 7, 9, 11], we have not demonstrated non‐inferiority of watchful waiting in this population. Confidence intervals that cross the non‐inferiority margin do not exclude the possibility that watchful waiting could result in more than 10 percentage points difference in Day 7 resolution compared with immediate antibiotics. Larger, adequately powered trials are required to determine whether watchful waiting is non‐inferior in urban Aboriginal and Torres Strait Islander communities. These findings cannot be extended to Aboriginal and Torres Strait Islander children living in remote areas, where risk of complications is much higher.

## Author Contributions

Jennifer Reath, Hasantha Gunasekera: conceptualisation, formal analysis, funding acquisition, investigation, methodology, project administration, resources, supervision, validation, visualisation, writing (original draft), writing (review and editing). Sanja Lujic: conceptualisation, formal analysis, data curation, investigation, methodology, resources, software, validation, visualisation, writing (original draft), writing (review and editing). Amanda J. Leach: conceptualisation, formal analysis, funding acquisition, investigation, methodology, supervision, validation, visualisation, writing (original draft), writing (review and editing). Letitia Campbell (Wiradjuri): conceptualisation, investigation, methodology, project administration, resources, supervision, validation, visualisation, writing (review and editing). Robyn Walsh: conceptualisation, data curation, investigation, methodology, project administration, resources, software, supervision, validation, writing (review and editing). Tim Usherwood: conceptualisation, formal analysis, investigation, methodology, supervision, validation, writing (review and editing). Geoffrey K. Spurling: conceptualisation, investigation, methodology, resources, validation, writing (review and editing). Claudette A. Tyson (Kuku Yalanji): conceptualisation, investigation, methodology, project administration, resources, supervision, validation, writing (review and editing). Deborah A. Askew: conceptualisation, methodology, resources, supervision, validation, writing (review and editing). Kelvin Kong (Worimi): conceptualisation, funding acquisition, methodology, resources, supervision, validation, writing (review and editing). Chelsea J. Watego (Mununjali Yugambeh and South Sea Islander): conceptualisation, funding acquisition, methodology, resources, supervision, validation, writing (review and editing). Peter Morris: conceptualisation, formal analysis, funding acquisition, methodology, resources, supervision, validation, writing (review and editing). Wendy Hu: conceptualisation, methodology, validation, visualisation, writing (review and editing). Penelope A. Abbott: conceptualisation, formal analysis, funding acquisition, investigation, methodology, project administration, resources, supervision, validation, visualisation, writing (original draft), writing (review and editing).

## Funding

This project was funded by a National Health and Medical Research Council Project Grant (#1046266 2014–2024; $1.6 million) and by Western Sydney University (Ainsworth Foundation; $250,000). The funders had no role in study design, data collection, analysis or interpretation, reporting or publication of this article.

## Disclosure

Not commissioned; externally peer reviewed.

## Conflicts of Interest

The authors declare no conflicts of interest.

## Supporting information


**Table S1:** Reporting against guidelines for reporting of non‐inferiority and equivalence randomised trials: an extension of the CONSORT statement.
**Table S2:** Watchful waiting compared with immediate antibiotics for urban Aboriginal and Torres Strait Islander children with uncomplicated acute otitis media (WATCH): a non‐inferiority randomised controlled trial. Study assessment schedule.
**Table S3:** Reporting according to CONSIDER Statement Huria T, Palmer SC, Pitama S, et al. Consolidated criteria for strengthening reporting of health research involving Indigenous peoples: the CONSIDER statement. BMC Med Res Methodol 2019;19:173.
**Table S4:** Subgroup, sensitivity and post hoc analyses.

## Data Availability

The de‐identified data we analysed are not publicly available, but requests to the corresponding author for the data will be considered on a case‐by‐case basis.
