# Supplementary material for: Watchful Waiting Compared With Immediate Antibiotics for Urban Aboriginal and Torres Strait Islander Children With Uncomplicated Acute Otitis Media (WATCH): A Non‐Inferiority Randomised Controlled Trial
Source: Med J Aust. 2026 Aug 13;224(8):e70260. doi: 10.5694/mja2.70260 (PMC13470832; doi:10.5694/mja2.70260)
Supplement: Supplementary file 1 [file MJA2-224-0-s001.pdf]

The Medical Journal of Australia

Supporting Information

Supplementary material

This appendix was part of the submitted manuscript and has been peer reviewed.
It is posted as supplied by the authors.

Appendix to: J. S. Reath, H. Gunasekera, S. Lujic, et al. Watchful Waiting Compared with Immediate Antibiotics for Urban Aboriginal and Torres Strait Islander Children with Uncomplicated Acute Otitis Media (WATCH): A Non-Inferiority Randomised Controlled Trial. *Medical Journal of Australia* 2026; doi: 10.5694/mja2.00000.

TABLE S1. Reporting against guidelines for reporting of noninferiority and equivalence randomized trials: an extension of the CONSORT statement

Piaggio G, Elbourne DR, Altman DG, Pocock SJ, Evans SJ; CONSORT Group. Reporting of noninferiority and equivalence randomized trials: an extension of the CONSORT statement. JAMA. 2006 Mar 8;295(10):1152-60. doi: 10.1001/jama.295.10.1152. Erratum in: JAMA. 2006 Oct 18;296(15):1842. PMID: 16522836.

Requirement	Evidence	Comment
1a. Title needs to include statement of non-inferiority design	✓	P 1
1 b. **Abstract:**<br>**Trial design**<br>**Methods**<br>Participants, Interventions intended for each group<br>Objective, Outcome<br>Randomisation, Blinding<br>**Results**<br>Numbers randomised, Recruitment (trial status)<br>Numbers analysed, Outcomes, Harms<br>**Conclusions**<br>**Trial registration**<br>**Funding**	✓	P1-2<br>Note: Funding is declared on p 12 of the paper and is not included in the abstract due to tight word count.
**2. Background and Objectives**<br>2a. Scientific background and explanation of rationale – rationale for using a noninferiority design<br>2b. Specific objectives or hypotheses including hypotheses concerning noninferiority specifying the noninferiority margin and the rationale for its choice.	✓	Included in Introduction<br>P 3
**3. Trial Design**<br>3a. Description of trial design<br>3b. Any important changes after commencement and rationale for these	✓	Trial design noted P3<br>Changes to age of recruitment and no involvement of practice nurses noted under participants p4
**4. Participants**<br>4a. Eligibility criteria for participants – whether those participating in the noninferiority trial are similar to those in any trials establishing the efficacy of the reference treatment<br>4b. Settings and locations where data were collected	✓	Eligibility, settings and locations described p 4<br>Studies describing the efficacy of watchful waiting are referenced in the introduction. No non-inferiority trials of the intervention have been conducted in this setting.

**5. Interventions**<br>The interventions for each group with sufficient details to allow replication, including how and when they were actually administered - Whether the reference treatment in the noninferiority trial is identical (or very similar) to that in any trial(s) that established efficacy	✓	P5<br>Watchful waiting is referenced in the introduction
**6. Outcomes**<br>6a. Completely defined prespecified primary and secondary outcome measures, including how and when they were assessed - Specify the noninferiority outcome(s) and whether hypotheses for main and secondary outcome(s) are noninferiority or superiority. Whether the outcomes in the noninferiority trial are identical (or very similar) to those in any trial(s) that established efficacy of the reference treatment<br>6b. Any changes to outcomes after the trial commenced with reasons	✓	6a Outcomes, hypotheses and analyses are documented on p5-7. The evidence for previous trials is summarised briefly in Reference 3, cited in Intro paragraph 1 and in the Discussion, but this work is mainly in high income countries and only from non-Indigenous children.<br><br>6b Change to age of recruitment noted under participants on p4
**7. Sample size**<br>7a. How sample size was determined - Whether the sample size was calculated using a noninferiority criterion and, if so, what the noninferiority margin was<br>7b. When applicable, explanation of any interim analyses and stopping guidelines - To which outcome(s) they apply and whether related to a<br>noninferiority hypothesis	✓	P5
**8. Sequence generation**<br>8a. Method used to generate the random allocation sequence<br>8b. Type of randomization; details of any restriction (such as blocking and block size)	✓	P6
**9. Allocation concealment mechanism**<br>Mechanism used to implement the random allocation sequence (such as sequentially numbered containers),describing any steps taken to conceal the sequence until interventions were assigned	✓	P6<br>(though allocation sequence was blinded, the allocation was not blinded other than for the blinded review sub-sample)
**10. Implementation**	✓	P6

Who generated the random allocation sequence, who enrolled participants, and who assigned participants to interventions		
**11. Blinding**	Not applicable	See above
**12. Statistical methods**<br>12a. Statistical methods used to compare groups for primary and secondary outcomes - Whether a 1- or 2-sided confidence interval approach was used<br>12b. Methods for additional analyses, such as subgroup analyses and adjusted analyses	✓	Pps 6-7
**13. Participant flow**<br>13a. For each group, the numbers of participants who were randomly assigned, received intended treatment, and were analyzed for the primary outcome<br>13b. For each group, losses and exclusions after randomization, together with reasons	✓	Figure 1
**14. Recruitment**<br>14a. Dates defining the period of recruitment and followup<br>14b. Why the trial ended or was stopped	✓	P4 notes dates of screening and P7 notes recruitment dates and why the trial ended
**15. Baseline data**<br>A table showing baseline demographic and clinical characteristics for each group	✓	P 18 (Table 2)
**16. No. analysed**<br>For each group, number of participants (denominator) included in each analysis and whether the analysis was by original assigned groups	✓	Primary and secondary analyses (both modified intention to treat and per protocol) are presented P 8 and Table 3 (pps19-20). Denominators are explained
**17. Outcomes and estimation**<br>For each primary and secondary outcome, results for each group, the estimated effect size, and its precision (such as 95% confidence interval) - For the outcome(s) for which noninferiority was hypothesized, a figure showing confidence intervals and the noninferiority margin may be useful<br>For binary outcomes, presentation of both absolute and relative effect sizes is recommended	✓	Reported p8 and Table 3 p19-20

**18. Ancillary analyses**<br>Results of any other analyses performed, including subgroup analyses and adjusted analyses, distinguishing prespecified from exploratory	✓	Sensitivity, sup-group and post hoc analyses are presented on pps 8-9 and in Appendix 4
**19. Harms**<br>All important harms or unintended effects in each group (for specific guidance see CONSORT for harms Ref 26 in Piaggio paper)	✓	P9 and also Pps 19-20 (Table 3)
**20. Limitations**<br>Trial limitations, addressing sources of potential bias, imprecision, and, if relevant, multiplicity of analyses	✓	Pps11-12
**21. Generalizability**<br>Generalizability (external validity, applicability) of the trial findings	✓	Heading removed at Editor request but information included Pps 11-12
**22. Interpretation**<br>Interpretation consistent with results, balancing benefits and harms, and considering other relevant evidence - Interpret results in relation to the noninferiority hypothesis- If a superiority conclusion is drawn for outcome(s) for which noninferiority was hypothesized, provide justification for switching	✓	Conclusions includes Interpretation but at Editor request this has been removed from the heading P12
**23. Registration**<br>Registration number and name of trial registry	✓	Noted p2 and p3
**24. Protocol**<br>Where the full trial protocol can be accessed, if available	✓	Appended to the submission also publication referenced at number 16
**25. Funding**	✓	P12

TABLE S2. Watchful waiting compared with immediate antibiotics for urban Aboriginal and Torres Strait Islander children with uncomplicated acute otitis media (WATCH): a non-inferiority randomised controlled trial
Study assessment schedule

Measure	Performed by	Day 0	Day 3[a]	Day 7[a]	Day 14[a]	Week 7[a]	3 months
Clinical assessment	General practitioner	X		X			
Temperature	General practitioner/ Research officer	X		X			
Tympanometry	General practitioner/ Research officer	X		X			
Digital Pneumatic Video-otoscopy	General practitioner/ Research officer	X		X		X	
Health history and Socio-demographics	Research officer	X					
Symptoms Scales[b]	Research officer	X	X	X	X		
Progress Questions	Research officer		X	X	X	X	
Serious Adverse Events/severe Study Related Adverse Events	Research officer/ Site-based investigator		X	X	X	X	
Structured medical record review	General practitioner						X
Blinded review of matched Pneumatic Video-otoscopy traces and tympanometry results	Otolaryngologist	X		X		X	

[a] Acceptable time windows: Day 3 (2-4); Day 7 (5-10); Day 14 (11-17) and Week 7 (6-8)

[b] Two symptom scales were used: Acute Otitis Media Severity of Symptoms (AOM-SOS) scale; and AOM-Faces scale.[37]

TABLE S3. Reporting according to CONSIDER Statement

Huria T, Palmer SC, Pitama S, et al. Consolidated criteria for strengthening reporting of health research involving Indigenous peoples: the CONSIDER statement. *BMC Med Res Methodol* 2019;19:173.

	Criterion	Evidence for consideration
	**Governance**	
1.	Describe partnership agreements between the research institution and Indigenous-governing organization for the research, (e.g., Informal agreements through to MOU (Memorandum of Understanding) or MOA (Memorandum of Agreement)).	Research contracts were signed with all sites
2.	Describe accountability and review mechanisms within the partnership agreement that addresses harm minimization.	Most sites established reference groups with First Nations community members providing input to the research team. Funding for these was detailed in the research contracts.<br>Site based research officers (majority identified First Nations) met regularly with the research manager and with the research team. Their input was sought and valued at all times and funding for related travel was detailed in the research contracts.<br>Opportunities were provided for First Nations only discussions with identified CIs
3.	Specify how the research partnership agreement includes protection of Indigenous intellectual property and knowledge arising from the research, including financial and intellectual benefits generated (e.g., development of traditional medicines for commercial purposes or supporting	According to the research contracts signed with each site, background intellectual property of the sites remained their property. Knowledge generated by the research could be used by sites although the intellectual property belonged to the institution

	the Indigenous community to develop commercialization proposals generated from the research).	sponsoring the research (Western Sydney University).<br>No commercialisation was envisaged as a result of this research.
	**Prioritization**	
4.	Explain how the research aims emerged from priorities identified by either Indigenous stakeholders, governing bodies, funders, non-government organization(s), stakeholders, consumers, and empirical evidence	Initially the research question was discussed with the CEO of one of the sites. A research team including experienced First Nations researchers developed the research proposal and sites were invited to comment on this prior to the application for research funding.
	**Relationships (Indigenous stakeholders/participants and Research team)**	
5.	Specify measures that adhere and honor Indigenous ethical guidelines, processes, and approvals for all relevant Indigenous stakeholders, recognizing that multiple Indigenous partners may be involved, e.g., Indigenous ethics committee approval, regional/national ethics approval processes.	The lead Ethics Committee for this project was the Aboriginal Health and Medical Research Council of NSW Ethics Committee (AHMRC HREC). Our application to this HREC fulfilled their requirements and we adhered to these throughout the research. AHMRC HREC have approved this publication for submission.
6.	Report how Indigenous stakeholders were involved in the research processes (i.e., research design, funding, implementation, analysis, dissemination/recruitment).	Staff and management of the Aboriginal Medical Services (mostly ACCHSs), and First Nations researchers on the research team contributed to research design, the funding application, implementation of the research, dissemination and recruitment. This is described in detail in the Trial Protocol and has been discussed in a qualitative review of the trial processes (both published and referenced in this paper at Refs 16 and 33)

7.	Describe the expertise of the research team in Indigenous health and research.	In addition to experienced First Nations researchers, our CI team included non-Indigenous researchers with a strong track record in respectful engagement in research with Aboriginal communities. Several had also worked as clinicians in Aboriginal Community Controlled Health Services.
	**Methodologies**	
8.	Describe the methodological approach of the research including a rationale of methods used and implication for Indigenous stakeholders, e.g., privacy and confidentiality (individual and collective)	Engagement with Aboriginal and Torres Strait Islander participants throughout the trial was led by local community research officers (mostly First Nations identified) employed by the Aboriginal Medical Services and adhered at all times to their protocols for privacy and confidentiality.
9.	Describe how the research methodology incorporated consideration of the physical, social, economic and cultural environment of the participants and prospective participants. (e.g., impacts of colonization, racism, and social justice). As well as Indigenous worldviews.	At all times we considered physical (where, culturally safe space), social (local community employment), economic (compensation) and cultural environments of participants and potential participants. This was led by the Aboriginal Medical Services with which we partnered and by the site based research officers appointed to the role by the Aboriginal Medical Services. Additionally at most sites a Research Reference Group advised on conduct of the research.<br><br>Site agreements ensured economic support for the AMS, for the employment of staff, the support for equipment, travel and compensation for the research, research staff and participants
	**Participation**	
10.	Specify how individual and collective consent was sought to	Consent was sought by either GPs or research officers employed by the

	conduct future analysis on collected samples and data (e.g., additional secondary analyses; third-parties accessing samples (genetic, tissue, blood) for further analyses).	Aboriginal Medical Services. Verbal explanations were given and written material and opportunities for questions were also provided. No additional analysis was planned therefore no permission was sought for this.<br><br>Consent was received from the AMS boards prior to commencement<br><br>Re-iterative checks occurred regularly with First Nation research officers and CIs to ensure methodology and analysis was appropriate.
11.	Described how the resource demands (current and future) placed on Indigenous participants and communities involved in the research were identified and agreed upon including any resourcing for participation, knowledge, and expertise	Funding was provided for the salary of the research officer and for meetings of the site based reference groups as well as for equipment required for the trial (which was also available for more general use in the Aboriginal Medical Service). Training for researcher officers was also provided. At all times we respected the need for clinical duties and responsibilities to take priority over the research.
12.	Specify how biological tissue and other samples including data were stored, explaining the processes of removal from traditional lands, if done, and of disposal.	Data were stored on site and in the client clinical records according to AHMRC HREC and NHMRC requirements.
	**Capacity**	
13.	Explain how the research supported the development and maintenance of Indigenous research capacity (e.g., specific funding of Indigenous researchers).	Please see above – salaries of all site based research officers were funded by the research and attendance at research meetings was also funded. Research officers were encouraged and supported in preparing and giving presentations including at international research meetings. Two Indigenous research officers were supported to devise research questions, set up their own studies, manage the project and then

		publish the data. All research officers meeting authorship criteria (including the two mentioned), have co-authored other WATCH publications to date.
14.	Discuss how the research team undertook professional development opportunities to develop the capacity to partner with Indigenous stakeholders?	As non-Indigenous researchers, we sought feedback regularly from First Nations research team members and participated in researcher development sessions led by these valued colleagues.
	**Analysis and interpretation**	
15.	Specify how the research analysis and reporting supported critical inquiry and a strength-based approach that was inclusive of Indigenous values.	At research team meetings including First Nations CIs and a number of the research officers both qualitative and quantitative data was reviewed and analyses and reporting agreed. First Nations team members were valued co-authors on all our publications and contributed extensively including through their own research (see above).
	**Dissemination**	
16.	Describe the dissemination of the research findings to relevant Indigenous governing bodies and peoples.	AHMRC HREC and participating sites have received a copy of this paper and prior to that a summary of the research outcomes was circulated to participating sites. A presentation from the research team was offered and where this was accepted the research findings were presented to Reference Groups and/or management.
17.	Discuss the process for knowledge translation and implementation to support Indigenous advancement (e.g., research capacity, policy, investment).	We have supported research officers at each site to present research findings, contribute at research meetings, assist with team publications and for some to publish their own research papers.<br><br>Our qualitative analysis of the trial process (Ref 33) provides recommendations for working with Aboriginal and Torres Strait Islander

		communities based on key guidelines by First Nations authors and based our learning throughout this research. The current paper re-iterates the importance of respectful partnership approaches to research in this area and the value such partnerships bring to non-Indigenous researchers.

TABLE S4. Subgroup, sensitivity and post hoc analyses

	Watchful waiting	Immediate Antibiotics	Absolute difference between watchful waiting and antibiotic arms Percentage points (90% CI[a])	P *(for non-inferiority)*
Modified intention-to-treat analysis data	106/128 (82.8%)	113/126 (89.7%)		
Sub-group analyses[b]				
Excluding three children retrospectively classified as ineligible (perforations on blinded review)	57/106 (53.8%)	67/110 (60.9%)	-7.1 (-18.2 to 3.9)	0.33
Excluding eight children retrospectively classified as ineligible (three above and four with baseline type A Tympanograms	56/105 (53.3%)	65/107 (60.7%)	-7.4 (-18.6 to 3.7)	0.35
Sensitivity analyses based on intention-to-treat cohorts				
Assuming no resolution for missing visits	57/128 (44.5%)	68/126 (54.0%)	-9.4 (-19.7 to 0.8)	0.46
Assuming resolution for missing visits	79/128 (61.7%)	81/126 (64.3%)	-2.6 (-12.5 to 7.3)	0.11
Assuming resolution for missing visits in Watchful Waiting arm and no resolution for those in Antibiotic arm	79/128 (61.7%)	68/126 (54.0%)	**7.8 (-2.4 to 17.9)**	**0.002**
Assuming no resolution for missing visits in Watchful Waiting arm and resolution for those in Antibiotic arm	57/128 (44.5%)	81/126 (64.3%)	-19.8 (-30.0 to -9.5)	0.94
**Numbers with Per-protocol analysis data**	97/128 (75.8%)	112/126 (88.9%)		
**Sensitivity analyses**				
Assuming no resolution for missing visits	49/119 (41.2%)	67/123 (54.5%)	-13.3 (-23.8 to -2.8)	0.70
Assuming resolution for missing visits	71/119 (59.7%)	78/123 (63.4%)	-3.8 (-14.0 to 6.5)	0.16
Assuming resolution for missing visits in Watchful Waiting arm and no resolution for those in Antibiotic arm	71/119 (59.7%)	67/123 (54.5%)	**5.2 (-5.2 to 15.6)**	**0.008**
Assuming no resolution for missing visits in Watchful Waiting arm and resolution for those in Antibiotic arm	49/119 (41.2%)	78/123 (63.4%)	-22.2 (-32.8 to -11.7)	0.97
Post-hoc analyses [b]				
Using Hoberman's[21] definition of resolution (AOM-SOS[c] score of 0, 1)	74/108 (68.5%)	98/116 (84.5%)	-16.0 (-25.2 to -6.8)	0.86
**Unilateral acute otitis media**[d]				

General practitioner determined resolution	29/50 (58.0%)	35/53 (66.0%)	-8.0 (-23.7 to 7.6)	0.42
**Bilateral acute otitis media cases** [d]				
General practitioner determined resolution	27/55 (49.1%)	33/60 (55.0%)	-5.9 (-21.2 to 9.4)	0.33
**Cases with more symptoms (Day 0 AOM-SOS[c] score ≥6)**				
General practitioner determined resolution	23/38 (60.5%)	26/39 (66.7%)	-6.1 (-24.1 to 11.8)	0.36
**Cases with less symptoms (Day 0 AOM-SOS[c] score <6)**				
General practitioner determined resolution	32/66 (48.5%)	41/73 (56.2%)	-7.7 (-21.6 to 6.2)	0.39

[a] Confidence Interval

[b] Denominators include only children for whom data were available at the relevant timepoint

[c] Acute Otitis Media – Severity of Symptoms Version 4

[d] Laterality of acute otitis media was not recorded for one child in the watchful waiting arm
